# Utilization of modern contraceptives among female health care workers at Gulu university teaching hospitals in Northern Uganda

**DOI:** 10.1186/s40834-024-00274-y

**Published:** 2024-04-06

**Authors:** Keneth Opiro, Jimmy Opee, Margret Sikoti, Pebolo Francis Pebalo, Jackline Hope Ayikoru, Harriet Akello, Priscilla Manano, Felix Bongomin

**Affiliations:** 1 Gulu University, Gulu, P. O. Box 166, Uganda; 2 St Mary’s Hospital Lacor, Gulu, P. O. Box 180, Uganda

**Keywords:** Modern contraceptives, Utilization, Healthcare workers, Gulu University

## Abstract

**Background:**

The global high rate of unintended pregnancy is a direct result of underutilization of contraceptives methods. Healthcare workers (HCWs) play a pivotal role in promoting and facilitating access to modern family planning services. By examining the extent to which healthcare providers practice what they preach, this research aimed to shed light on the prevalence and factors associated with modern contraceptive use among female HCW at two university teaching hospitals in northern Uganda.

**Methods:**

A cross-sectional survey was conducted among qualified female healthcare workers (FHCWs) at Gulu Regional Referral Hospital (GRRH) and St. Mary’s Hospital-Lacor in Gulu, Uganda. Convenient consecutive sampling was used to enroll study participants. Linear regression analysis was employed to determine factors independently associated with modern contraceptive use. *P* < 0.005 was considered statistically significant.

**Results:**

We enrolled 201 female HCWs, with a median age 31 (interquartile range: 27–38) years. Overall, 15 (7.5%, 95% Confidence Interval [CI]: 4.4 —11.1) participants utilized modern methods of family planning in the last 3 months while lifetime use was at 73.6% (*n* = 148, 95%CI: 67.3 — 79.4%). Most common method utilized was intra-uterine devices [IUDs] (51%, *n* = 76), followed by sub-dermal implants (15.4%, *n* = 23). Eighty-five (42.3%, *n* = 85) participants had desire to get pregnant. Factors independently associated with utilization of modern methods contraceptives were working at GRRH (adjusted odds ratio (aOR): 5.0, 95% CI: 1.59 — 10.0, *p* = 0.003), and being single (aOR: 3.3, 9%CI: 1.02 —10.57, *p* = 0.046).

**Conclusions:**

Utilization of modern methods of contraceptive among female HCWs in this study is lower than the Uganda national estimates for the general female population. Most utilized method is IUDs followed by sub-dermal implants. More studies are recommended to see if this finding is similar among FHCWs in other regions of Uganda and the rest of Africa while also considering Male Healthcare Workers.

## Background

**Fig. 1:**
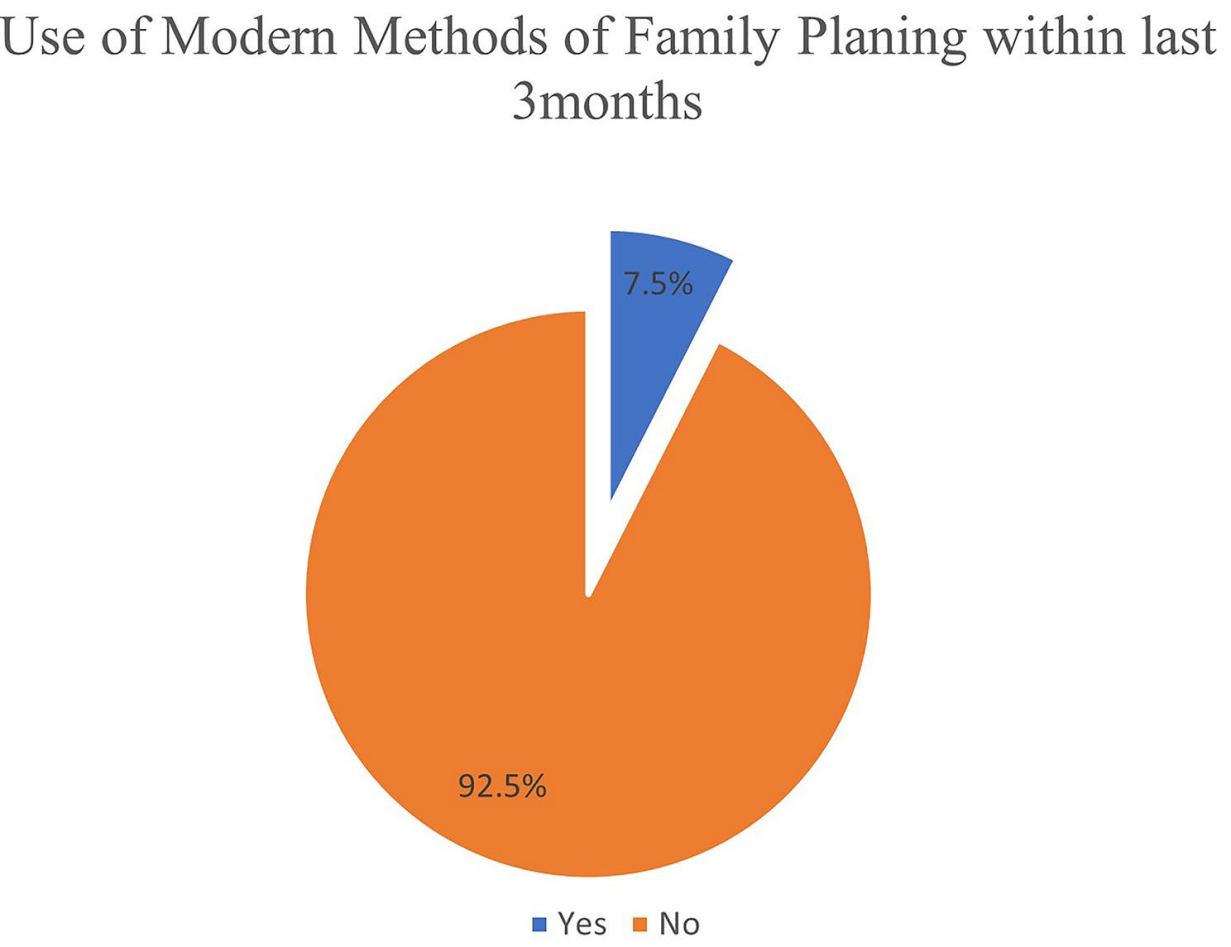
Utilization of modern methods of family planning within last 3 months

Over 121 million unintended pregnancies occur annually among women of reproductive age globally, out of which 61% end in abortion with Uganda having a 3-fold higher rates of unintended pregnancy, where 145(131 to 159) per 1000 women of reproductive age (15–49 years) have unintended pregnancies compared to global figures [1]. Globally, an estimated 270 million women have unmet needs for modern contraceptive methods [2], and the figure is disproportionally high for women in low-income countries (214 million women) [3]. Despite the increased awareness and utilization of modern methods of family planning (FP), unmet need of family planning has remained high in developing countries [3–5] with about 1 in 4 women in sub–Saharan Africa having an unmet need [6].

In Germany, 37.2% of female gynecologists reported personally using Combined Oral contraceptives [7]. A cross-sectional survey done in Ghana among healthcare workers and clinical year medical students on attitudes and practice of contraceptives among 400 study participants, showed that among the qualified healthcare workers, more than half of the participants (62.4%) had used a form of family planning although only 1 in 5 (18%) were actively using it at the time of survey, and condoms and other barrier methods were most preferred [8].

In Uganda, we could not find any published study which assessed the prevalence of FP use among healthcare workers (HCWs) especially in the context of northern Uganda. However, in other population groups, the prevalence of utilization of modern contraceptives varies with different studies assessing different groups. Prevalence was found to be low among adolescent females where only 9.4% (401/4264) were found to be using modern methods using Uganda Demographic and Health Survey (UDHS) dataClick or tap here to enter text., over 24% of girls aged between 15 and 19 are already having children and 28% of women had unmet need for FP [9, 10]. Prevalence of contraceptive use among adolescent refugees in northern Uganda was reported at 8.7% (73/839) and 36% (155/434) among women living with HIV attending services at Gulu Regional Referral Hospital (GRRH) in northern Uganda [11, 12].

Several factors have been shown to influence choice of use of modern methods of contraceptives among women of reproductive age. A systematic review analyzing factors influencing contraceptive use in sub-Saharan Africa found negative factors reducing the use to be misconception about side effects, male partner disapprovals and social/cultural influence while positive factors included education, employment, and effective communication between partners [13]. Other studies in other settings showed similar findings [13–17]. Similarly, in Uganda, distance to health facility, knowledge, geographic locations, age, education level, religion, employment, and number of children were all found to be factors that influence utilization of modern methods of contraceptives [9, 18–20]. All these studies considered female population with hardly any study done among female healthcare workers especially in the context of northern Uganda.

A study done to compare contraceptive use among female HCWs and females in the general population aged 23 to 49 years in Spain found that Condoms were the most widely used methods in both groups and the reason cited for this was fear of side effects and female HCWs preferred long-acting reversible methods than oral contraceptive as compared to their counterparts in the general population [21]. HCWs are on the frontline for advocating and delivering modern contraceptive methods of family planning and it is possible that clients themselves ask experiences of healthcare workers if they are also using it. However, as the saying goes “…*live by example*”; are HCWs really practicing these methods and which factors influence their use? Despite HCWs being at the frontline, the prevalence and factors associated with modern methods contraceptive use among them in northern Uganda is not documented.

Understanding prevalence and factors associated with modern methods of contraceptive in this setting can be useful in identifying modifiable factors and hence significant in addressing modalities to increasing utilization of contraceptives among them which might indirectly influence their attitudes, also known as provider bias, towards provision of this service to the general population [22]. Therefore, this study aimed at assessing the prevalence and factors associated with utilization of modern methods of contraceptives among female HCWs at the two Gulu University Teaching Hospitals in northern Uganda.

## Study methods

### Study design

We conducted a cross-sectional survey among female HCWs between January and May 2023 at GRRH and St. Mary’s Hospital -Lacor, Gulu, Uganda.

### Study settings and population

The study was conducted among professional female HCWs working in the two Gulu University teaching hospitals: GRRH and St. Mary’s hospital Lacor (LH), both in Gulu city, Uganda. Gulu City is found in the Northern part of Uganda, about 360 km from Kampala capital city. Qualified professional healthcare workers were defined as workers who have studied formal professional health courses with a minimum of certificate such as nurses, midwives, health educators, doctors, among others. Gulu city has two divisions; Bardege-Layibi (where LH is located) and Pece-Laroo (where GRRH is locatd) Divisions. GRRH is a public hospital that offers free services to the community and also has a family planning unit providing a wide range of modern methods which include: Implants, Injectables, Intrauterine Contraceptive Device (IUCD), sterilization (tubal/ligation), Combined Oral Contraceptive Pills (COC), Condoms, and emergency contraceptive pills. The unit records average monthly utilization by clients of minimum (personal communication with Nurse in-charge of FP unit). Meanwhile LH is a private not for profit healthcare facility, funded by the Catholic Church. It doesn’t directly offer Modern methods of FP except for few natural methods such as moon beads, but offers counselling and referring women for family planning, hence, the family planning service provision is not interrupted in the continuum of care. There are more than 1,000 healthcare workers in the two hospitals with female population slightly above 600 but only 405 professional female healthcare workers in both hospitals most of whom are trained in family planning.

### Sample size and sampling methods

Sample size was estimated using the Slovin’s formula for finite population where the female population of professional healthcare workers in the two hospitals were 405 and 5% absolute error limit, sample size of 201 was obtained. This sample size was divided equally between the two hospitals. In each hospital, sample size was divided between 6 main departments: Obstetrics and gynecology (including sexual and reproductive health and FP units), Surgery, Pediatrics, Outpatient, Emergency, and internal Medicine Departments, bringing sample size of 16–17 respondents per department. Consecutive sampling method was used in each department until sample size for that particular department was reached.

### Inclusion and exclusion criteria

Only female professional healthcare workers were included in the study. Male healthcare workers, none professional female HCWs such as drivers, cleaners, porters, security guards, records keepers, among others were excluded.

### Ethical considerations

The study protocol was approved by the Gulu University Research Ethic Committee (GUREC) (approval number: GUREC-2022-426). Administrative clearances were also obtained from hospital administrators of both hospitals. Participation in the study was voluntary from participants and informed written consent was mandatory before participating in the study. Participants were free to withdraw from the study at any one point even during the interview. Privacy was ensured by conducting the interviews in a side room one on one. There will be dissemination of study results in which the participating facilities and the study participants will be informed about the study results.

### Data collection and management

#### Data collection and tool

pre-tested researcher-administered questionnaires were used to collect data. To ensure the questions were understandable with ease, the questionnaire was pre-tested by directly administering to 10% of our sample size (*n* = 20) among FHCWs in these two hospitals (*n* = 10 for each hospital) and necessary corrections were made. These pre-test participants were not excluded in the sample size of the final study participants. Four female (female for ease of interactions with participants who were all female) research assistants, two for each hospital, were employed and trained in collecting the data. Study participants were offered brief explanation about the study, obtained voluntary written consent, and directly administered the questionnaires to the participants. Convenient consecutive sampling method was used until the required sample was reached. To ensure adequate time, participants were allowed to decide on time for the interview when they were free from active hospital duties.

Information obtained were sociodemographic data, sexual and reproductive history, and use of modern contraceptives.

### Data analysis

Data collected were entered into RedCap data base and kept in password protected computer only accessible to the study team. Data were exported and analyzed using STATA version 17. Dependent variable was; utilization of modern methods and independent variables were; Socio-demographic factors such as age, level of education, hospital station, religion, place of residence, marital status, number of children, desire to get pregnant, Working experience (years), working in family planning unit, training in FP services, change of partner and if ever advised colleagues to use modern contraceptives; and Obstetric factors such as parity, abortions and if ever had complications in previous pregnancy.

## Results

### Socio-demographic characteristic of the participants

We enrolled 201 participants, with median age of 31 (IQR:27–38) years and most (62.7%, *n* = 126) were of age less than 35 years old, catholic (60.2%, *n* = 121) and from Bardege-Layibi division (69.2%, *n* = 139). More than half of the participants were certificate holders (54.7%, *n* = 110), approximately half worked in family planning clinic (45.3%, *n* = 91) and had training on emergency contraceptive (49.2%, *n* = 99). Table 1.

**Table 1:** Sociodemographic characteristic of the participants and MCU

Variable	Frequency	Percentage
**Age, median(IQR), years**	**31**	**27–38**
< 35 ≥ 35	126 75	62.7 37.3
**Hospital station**		
GRRH Lacor	100 101	49.8 50.2
**Parity, median(IQR)**	**2**	**1–3**
Grand multiparous (≥ 5 pregnancies ≥ 28 weeks) Multiparous (≥ 2 pregnancies ≥ 28 weeks) Nulliparous (0 pregnancy ≥ 28 weeks) Prime parous (1 pregnancy ≥ 28 weeks)	13(6.5) 106(52.7) 35(17.4) 47(23.4)	6.5 52.7 17.4 23.4
**Working experience, median(IQR), years**	**5**	**3–9**
**Religion**		
Catholic Protestant Born again Moslem	121 48 30 2	60.2 23.9 14.9 1
**Education level**		
Certificate Degree Diploma Masters	110 21 69 1	54.7 10.5 34.3 0.5
**Marital status**		
Single Cohabiting Married Widow	33 47 119 2	16.4 23.4 59.2 1
**Residence**		
Bardege-layibi division Laroo-pece division	139 62	69.2 30.8
**Complication in the previous pregnancy**		
No yes	156 45	77.6 22.4
**Desire to get pregnant**		
No Yes	116 85	57.7 42.3
**Worked in family planning clinic?**		
No Yes	110 91	54.7 45.3
**Training on emergency contraceptive**		
No Yes	102 99	50.8 49.2
**Number of living children, median(IQR)**	**2**	**1–3**
**Ever Had abortion**		
No Yes	178 23	88.6 11.4
**Changed partner**		
No Yes	139 62	69.1 30.9
**Advised people to use MC**		
No Yes	25 176	12.4 87.7
**Last use of MC**		
0–3 months ago 3–5 years 4months to 2 years > 5 years Currently using	15 18 71 11 22	11 13.1 51.8 8 16.1

### Reproductive characteristics of the participants

The median parity of the participants was 2 (IQR:1–3) with median number of living children being 2 (IQR:1–3). Seventy seven%,77.6% (*n* = 156) had no complication in their previous pregnancy and less than half have desire to get pregnant (42.3%, *n* = 85), Table 1.

### Factors associated with utilization of modern methods of family planning

**Fig. 2:**
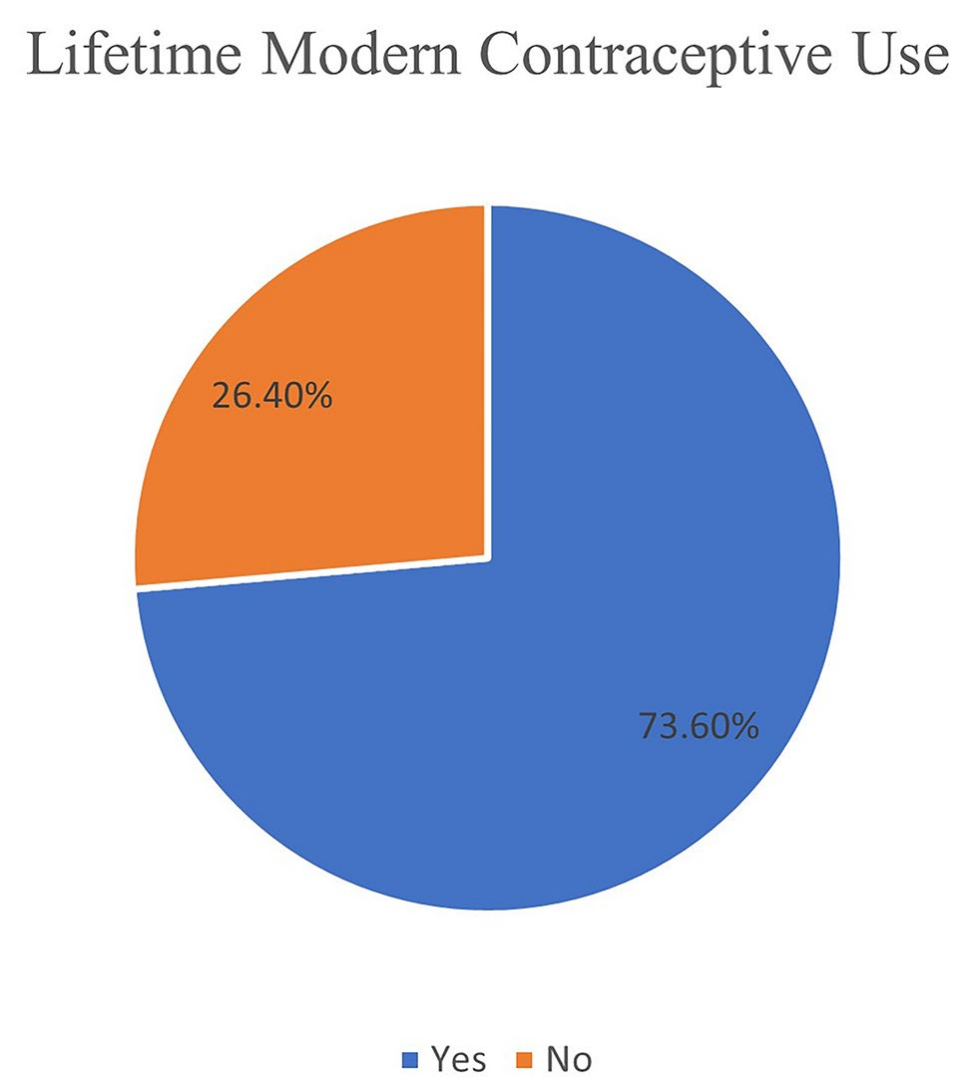
Lifetime utilization of modern methods of family planning

**Fig. 3:**
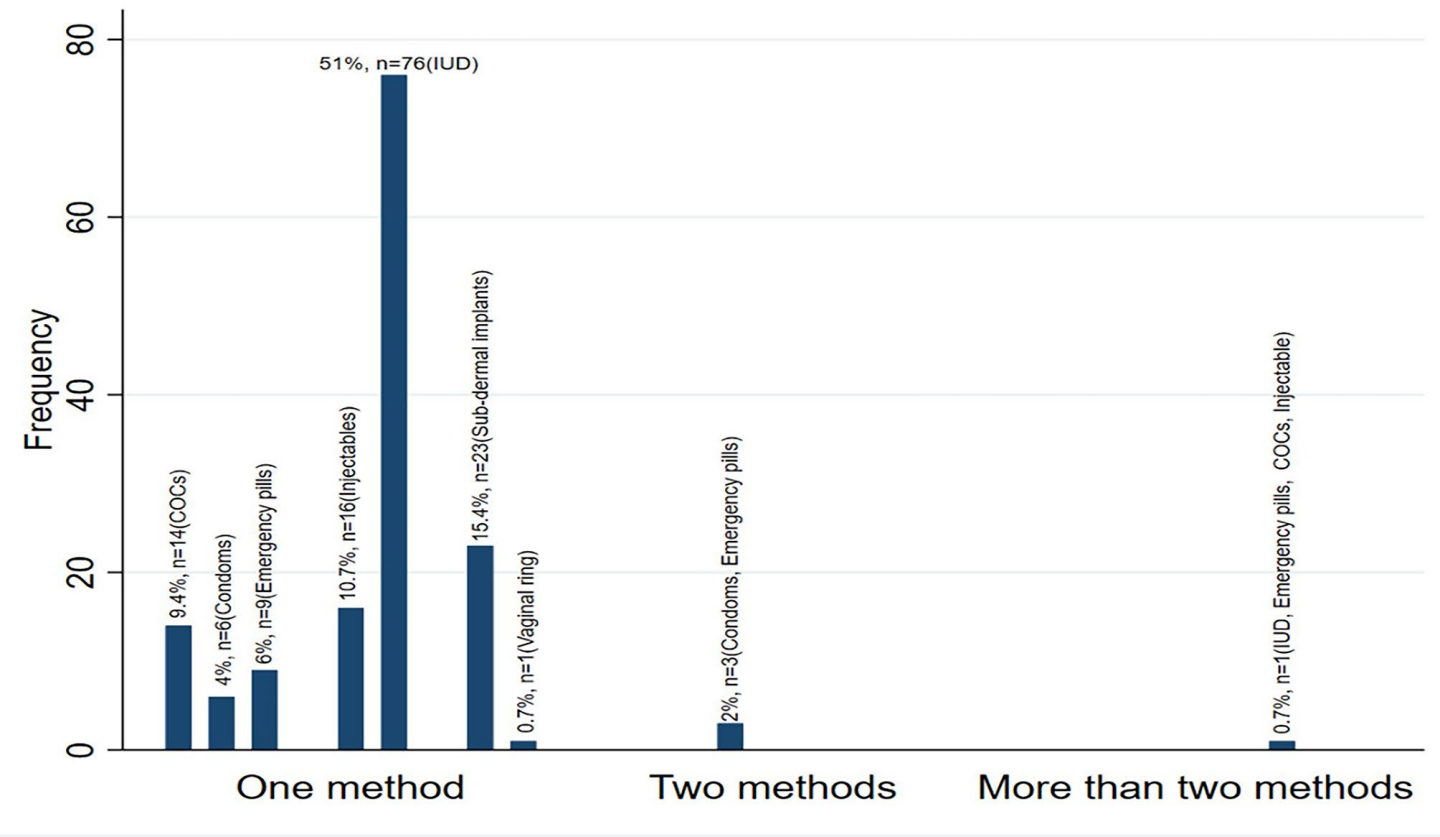
Difference contraceptive utilized

Utilization of modern methods of family planning within the last 3 months was at 7.5% (*n* = 15, 95% Confidence Interval [CI]: 4.4 —11.1) (*n* = 15) (Fig. 1) while lifetime use (ever used) was at 73,6%, *n* = 148 (Fig. 2) a. IUDs was the most utilized method (51%, *n* = 76), followed by sub-dermal implants (15.4%, *n* = 23) (Fig. 3).

At bivariate analysis, factors that were significantly associated with utilization of modern methods of family planning were age (*p* = 0.022), hospital station (*p* < 0.001), parity(*p* < 0.001), marital status(*p* < 0.001), residence(*p* = 0.015), ever worked in family planning clinic(*p* = 0.011), number of living children (*p* < 0.001) and if participants have ever advised people (friends, colleagues, those who seek their opinion outside professional work at FP unit) to use modern methods of family planning (*p* < 0.001), Table 2.

**Table 2:** Bivariate analysis for factors associating with utilization of modern methods of family planning

Variables	All (*N* = 201) Freq (%)	Modern methods of family planning lifetime use		*P* value
		Yes (*n* = 148) Freq (%)	No (*n* = 53) Freq (%)	
**Age**, median(IQR), years	31 (27–38)	32(28-38.5)	29 (26–36)	**0.022**
< 35 >=35	126(62.7) 75(37.3)	87(58.8) 61(41.2)	39(73.6) 14(26.4)	0.069
**Hospital station**				
GRRH Lacor	99(49.3) 102(50.7)	88(59.5) 60(40.5)	11(20.8) 42(79.3)	**< 0.001**
**Parity**, median(IQR)	2 (1–3)	2 (1–3)	1(0–2)	**< 0.001**
Grand multiparous Multiparous Nulliparous Prim parous	13(6.5) 106(52.7) 35(17.4) 47(23.4)	9(6.1) 92(62.2) 16(10.8) 31(21)	4(7.6) 14(26.4) 19(35.9) 16(30.2)	**< 0.001**
**Working experience**, Median(IQR), years	5 (3–9)	5 (3–9)	5 (2–10)	0.626
**Religion**				
Catholic Protestant Born again Moslem	121(60.2) 48(23.9) 30(14.9) 2(1)	93(62.8) 36(24.3) 18(12.2) 1(0.7)	28(52.8) 12(22.6) 12(22.6) 1(1.9)	0.247
**Education level**				
Certificate Degree Diploma Masters	110(54.7) 21(10.5) 69(34.3) 1(0.5)	78(52.7) 14(9.5) 55(37.2) 1(0.7)	32(60.4) 7(13.2) 14(26.4) 0(0)	0.455
**Marital status**				
Single Cohabiting Married widow	33(16.4) 47(23.4) 119(59.2) 2(1)	14(9.5) 36(24.3) 98(66.2) 0(0)	19(35.9) 11(20.8) 21(39.6) 2(3.8)	**< 0.001**
**Residence**				
Bardege-layibi division Laroo-pece division	139(69.2) 62(30.8)	95(64.2) 53(35.8)	44(83) 9(17)	**0.015**
**Complication in the previous pregnancy**				
No yes	156(77.6) 45(22.4)	116(78.4) 32(21.6)	40(75.5) 13(24.5)	0.702
**Desire to get pregnant**				
No Yes	116(57.7) 85(42.3)	81(54.7) 67(45.3)	35(66) 18(34)	0.195
**Worked in family planning clinic?**				
No Yes	110(54.7) 91(45.3)	73(49.3) 75(50.7)	37(69.8) 16(30.2)	**0.011**
**Training on contraceptive**				
No Yes	102(50.8) 99(49.2)	70(47.3) 78(52.7)	32(60.4) 21(39.6)	0.112
**Number of living children**, median(IQR)	2(1–3)	2(1–3)	1(0–2)	**< 0.001**
**Ever Had abortion**				
No Yes	178(88.6) 23(11.4)	132(89.2) 16(10.8)	46(86.8) 7(13.2)	0.622
**Changed partner**				
No Yes	139(69.1) 62(30.9)	99(66.9) 49(33.1)	40(75.5) 13(24.5)	0.300
**Advised people to use MC**				
No Yes	25(12.4) 176(87.7)	8(5.4) 140(94.6)	17(32.1) 36(67.9)	**< 0.001**

At multivariable analysis, Table 3. Factors independently associated with utilization of modern methods contraceptives were working at GRRH (adjusted odds ratio (aOR): 5.0, 95% CI: 1.59 — 10.0, *p* = 0.003), and being single (aOR: 3.3, 9%CI: 1.02 —10.57, *p* = 0.046).

**Table 3:** Multivariable logistic regression analysis for factors independently associated with utilization of modern method of family planning

Variable	Crude Odds ratio(95% CI)	*P*-value	Adjusted Odds ratio(95% CI)	*P*-value
**Age**				
< 35 >=35	Ref 2(0.98–3.91)	0.058	Ref 1.7(0.59–4.66)	0.343
**Hospital station**				
GRRH Lacor	Ref 0.2 (0.09–0.37)	< 0.001	5.0 (1.59-10.0) Ref	**0.003**
**Marital status**				
Single Cohabiting Married	4.4(1.69–11.67) 6.3(2.75–14.61) Ref	0.002 < 0.001	3.3(1.02–10.57) 2.8(0.87–9.18)	**0.046** 0.085
**Residence**				
Bardege-layibi division Laroo-pece division	Ref 2.7(1.23–6.02)	0.013	Ref 1.1(0.39–3.11)	0.851
**Worked in family planning clinic?**				
No Yes	Ref 2.4(1.22–4.64)	0.011	Ref 1.8(0.80–3.88)	0.851
**Training on emergency contraceptive**				
No Yes	Ref 1.7(0.89–3.21)	0.104	Ref 1.3(0.62–2.96)	0.454
**Parity**				
Grand multiparous Multiparous Nulliparous Prim parous	2.7(0.69–10.33) 7.8(3.26–18.64) Ref 2.3(0.94–5.64)	0.154 < 0.001 0.069	1.6(0.22–11.61) 3.4(0.92–12.26) Ref 1.8(0.54–5.77)	0.640 **0.066** 0.342

## Discussions

In this study which aimed at determining the level of utilization of modern contraceptive methods among female HCWs in two large teaching hospitals in Northern Uganda, despite nearly 3 in 4 of the respondents having a history of a lifetime use of at least one method of modern contraceptive, only 1 in 13 female HCWs were found to have used it in the last 3 months. There are no previous studies on the utilization of modern contraceptives among female HCWs in Uganda.

A cross-sectional survey done in Ghana among healthcare workers and clinical-year medical students (students who have completed basic sciences and now allowed to interact with patients/clients) on attitudes and practice of contraceptives among 400 study participants, showed that among the qualified healthcare workers, more than half of the participants (62.4%) had used a form of family planning although only 1 in 5 (18%) were actively using it at the time of survey, and condoms and other barrier methods were most preferred [8]. This shows higher prevalence for recent use in their study compared to our findings but the lifetime use in our study was higher than in their study. This could be possibly due to variations in study participants in Ghana where both males and females HCWs participated whereas in our study only female healthcare workers participated in the study. In Germany, 37.2% of female gynecologists reported personally using Combined Oral contraceptives [7]. This is higher than what we found in our study though their study population was restricted to gynecologists compared to our study.

Comparing the prevalence rates with the ones among the general female population, our study showed lower than the findings got among the general female population in Uganda based on data from Uganda Demographic Health Surveys whereby uptake of modern contraceptive improved from 11.6% in the year 1995 to 32.1% in 2011 [23]. Prevalence is also higher (36%) among HIV-positive women than the finding in this study, according to one study conducted among clients attending HIV clinics at Gulu Regional Referral Hospital [12]. This could be due to routine integration of contraceptive service in HIV clinics as one of the pillars in preventing mother to child transmission of HIV among HIV positive women [24]. Study among over 1,000 University female students in Uganda reported active contraceptive use at 46.6% with barrier methods being most preferred [25]. Similarly, prevalence is also higher among married Somali women living in Kampala (29%), among women living in in informal urban settlements in Kampala (47.4%), and among postpartum mothers in Uganda (28%) than the findings in our study [26–28]. And a comparative analysis using UDHS in 2011, of contraceptive prevalence among younger females of age 15–24 years was 34% and 50% among older women of age 25–34 years in the general female population both of which are still higher than the prevalence found in our study found [18]. Survey of modern contraceptive rates among women of reproductive age (15–49) in sub-Saharan Africa varied among females who are married or in union 45.7% [29], 26% among women in 20 African countries [30] and 17% in another study involving 17 countries sub-Saharan [31].

From various studies among female adults in Uganda and across Africa, prevalence is higher varying between 26 and 50%, among general female populations compared to female healthcare workers in our study. This could be due to the fact that healthcare workers are professionally trained and know the physiology of menstrual cycle and therefore are able to successfully apply traditional fertility awareness method instead of using modern contraceptives or they are simply not “walking the talk”. Exceptions are from younger female population groups where prevalence is lower than what we found in this study, for example, among female adolescent populations with only 9.4% and refugee adolescent population 8.7%, and across sub-Saharan Africa 24.7% [11, 32, 33]. This could be explained from the fact that young adolescents in Uganda generally from cultural perspective are forbidden from engaging in sexual activities, and even if some would engage in it, would do in disguise. Therefore, going for modern contraceptives would not be an easy to do task due to fear of perception of others and also advice from colleagues and family members [34, 35]. Also, studies have shown influence of provider bias on provision of modern contraceptives to women based on socio-demographic factors such as age with tendency to question effects of hormonal methods on fertility among younger population, which could also offer possible explanations on the low contraceptive use among younger population [22]. Comparing with global contraceptive rates, our finding is still lower than the global rates estimated in 2017 from the general female population [36].

Most common method used by the FHCWs found in this study was IUDs with more than half reported preferring it (51.0%, *n* = 76) followed by sub-dermal implants at 15.4%. This is similar with the study among health workers in Ghana where barrier method was preferred [8], but in contrast, the study comparing Spanish female healthcare workers and women in the general population found condoms being the most preferred in both groups [21]. Among females in the general population in Uganda, the most commonly used method according to Family Planning Atlas by UNFPA 2020 was injectables with every 1 in 5 women using this method [37]. Survey across sub-Saharan Africa also found most commonly used method among general female population of reproductive age being injectables at 39.4% followed by implants at 26.5% [29], with similar finding in a large population survey among 20 African countries [30]. This difference in the preferred method between female healthcare workers and general female population could possibly be due to the fact that healthcare professionals know much more on side effects of hormonal methods compared to barrier ones [38], ease of use of barrier methods, and the user can stop at any point without derailing hormonal cycle while resuming fertility instantly [21].

At bivariate analysis, our study showed the factors significantly associated with utilization of modern methods of family planning were; age, hospital station, parity, marital status, location of residence, ever worked in family planning clinic, number of living children, and ever having advised people to use modern methods of family planning. Although there is a significant variation across countries in how various factors; individual, community and service delivery, influence utilization of modern contraceptives [39], many factors are consistent across countries with findings in many studies carried out in Uganda [18, 26, 28, 32, 40], across Africa [13–15, 27, 18, 30, 31, 33, 41–44], and the rest of the globe [16, 17, 45].

At multivariate analysis, age 35 years and above, working in GRRH, having worked in FP clinics, being single or only cohabiting, having been trained on FP services and multiparity were positively associated with using contraceptives using crude odds ratios, however, after adjusting for confounders, only two factors had significant positive associations; Being Single had more than 3 times likelihood of using modern contraceptives compared to married women and female staff working at GRRH had five times likelihood of using modern contraceptives than those working in LH. Most likely explanation for working at GRRH being an independent and significant positive associated factors with staff using FP is possibly because whereas in GRRH there is operational FP clinic where the services are freely offered, in LH, being Roman Catholic Church founded [46, 47], there is no FP service being directly offered within the facility with exception of medically indicated methods such as tubal ligation during cesarean section of a multiparous woman or those with bad obstetric history, in that stopping subsequent pregnancy is considered a method to prevent future maternal complications. Therefore, HCWs could be indirectly influenced by the practice in this faith-based hospital, hence their low uptake.

Being single, was associated with three times likelihood of using modern contraceptives compared to married FHCWs or those cohabiting. This could be due to that fact that single women would not want to get pregnant before marriage or before having official partner which leads to becoming single mothers, hence need to use FP. This is true especially in the context of Uganda were getting pregnant before marriage is negatively perceived as a shame not only to the woman herself but also the family members and FHCWs are not exceptional when it comes to community perception although this has changed in recent years [48, 49].

Our study findings show that majority of FHCWs in these hospitals are not practicing what they preach. The utilization is lower than expected in comparison to other female population given the fact that these are HCWs who are in the frontline in advocating and delivering modern contraceptives and are very much aware of the importance. The implication of this to the lay population could be that, none healthcare workers might get discouraged and wonder why those delivering the service are not really using them and yet advocating and delivering the services. Furthermore, the methods used by the FHCWs are significantly different from the general population and this might also give different signals to none healthcare professionals and may play role provider-bias while counseling and delivering these services to clients. On a positive side, the finding could mean HCWs prefer IUDs and implants due to the fact that they have busy work schedules and have little time for pills or injectables indicating that they are well conversant with the methods and hence the choices.

### Strength and possible limitations of this study

Our study recruited up to half of female staff working in these two largest hospitals in northern Uganda, making the study generalizable to FHCWs in northern Uganda. Since this was individual staff response to interviewers, there may be desirability bias (staff reporting what is expected and desired by them rather than reality), however, the design of the questions was done in such a way as to eliminate desirability bias as much as possible.

There could have been hurried responses from staff due to busy work schedules. This was avoided as much as possible by allowing participants to decide on the time of interview when they were ready with adequate allocated time.

Researchers also sampled and paid direct observations on the data collection process being implemented by the interviewers.

Data processing and security in terms of encryption and use of password reduced likelihood of any third party editing the information.

Sample size is representative enough for the two hospitals in northern Uganda but may not be enough to represent the entire Uganda. There is further need to do this survey in other regions in Uganda.

## Conclusions

Prevalence of modern contraceptive use among female HCWs in Gulu District, northern Uganda is low compared to general population implying HCWs are not really up to about what they preach. Most commonly used method is IUDs in contrast to injectables among general population. Being single and working at GRRH were independent factors increasing the likelihood of FHCWs using modern contraceptives. We recommend further in-depth interviews to understand utilization of modern contraceptives among FHCWs in other regions of Uganda while including male HCWs and further advocacy among FHCWs to improve utilization in northern Uganda.

## Data Availability

The datasets used and/or analyzed during the current study are available from the corresponding author on reasonable request.

## Abbreviations

SDG: Sustainable Development Goal
UN: United Nations
WHO: World Health Organization
GUREC: Gulu University Research Ethics Committee
GUTH: Gulu University Teaching Hospitals
UNCST: Uganda National Council of Science and Technology
HIV: Human Immunodeficiency Virus
FP: Family Planning
FHCWs: Female Healthcare Workers
MC: Modern Contraceptives
MCU: Modern Contraceptive Use
IUCD: Intrauterine Contraceptive Device
COC: Combined Oral Contraceptives

## References

[1] Bearak J, Popinchalk A, Ganatra B, Moller AB, Tunçalp Ö, Beavin C (2020). Unintended pregnancy and abortion by income, region, and the legal status of abortion: estimates from a comprehensive model for 1990–2019. Lancet Glob Health.

[2] Kantorová V, Wheldon MC, Ueffing P, Dasgupta ANZ. Estimating progress towards meeting women’s contraceptive needs in 185 countries: a bayesian hierarchical modelling study. PLoS Med. 2020;17(2). 10.1371/journal.pmed.1003026 PMC7028249 32069289

[3] World. Health Organisation. Family Planning/Contraception Methods. 2021.

[4] Wulifan JK, Brenner S, Jahn A, De Allegri M (2015). A scoping review on determinants of unmet need for family planning among women of reproductive age in low and middle income countries. BMC Womens Health.

[5] Ewerling F, Victora CG, Raj A, Coll CVN, Hellwig F, Barros AJD (2018). Demand for family planning satisfied with modern methods among sexually active women in low-and middle-income countries: who is lagging behind?. Reprod Health.

[6] Kantorová V, Wheldon MC, Ueffing P, Dasgupta ANZ (2020). Estimating progress towards meeting women’s contraceptive needs in 185 countries: a bayesian hierarchical modelling study. PLoS Med.

[7] Wiegratz I, Galiläer K, Sänger N, Rody A, Kuhl H, Schleußner E (2010). Prescribing preferences and personal experience of female gynaecologists in Germany and Austria regarding use of extended-cycle oral contraceptives. Eur J Contracept Reproductive Health Care.

[8] Agbeno E, Osarfo J, BAFIJ. of, 2021 undefined. Attitudes and Practices of Healthcare Professionals and Clinical Medical Students on Contraception: A Cross-Sectional Study in Cape Coast, Ghana. hindawi.com [Internet]. [cited 2023 Jun 4]; Available from: https://www.hindawi.com/journals/ijrmed/2021/6631790/. 10.1155/2021/6631790 PMC8012129 33834058

[9] Sserwanja Q, Musaba MW, Mukunya D (2021). Prevalence and factors associated with modern contraceptives utilization among female adolescents in Uganda. BMC Womens Health.

[10] Kemigisha E, Bruce K, Nyakato VN, Ruzaaza GN, Ninsiima AB, Mlahagwa W (2018). Sexual health of very young adolescents in South Western Uganda: a cross-sectional assessment of sexual knowledge and behavior. Reprod Health.

[11] Bakesiima R.… AC, 2020 undefined. Modern contraceptive use among female refugee adolescents in northern Uganda: prevalence and associated factors. reproductive-health-journal… [Internet]. [cited 2023 Jun 2]; Available from: https://reproductive-health-journal.biomedcentral.com/articles/10.1186/s12978-020-00921-y. 10.1186/s12978-020-00921-y PMC7238518 32434523

[12] Bongomin F, Chelangat M, AEBR. 2018 undefined. Prevalence and factors associated with contraceptive use among HIV-infected women of reproductive age attending infectious disease clinic at Gulu Regional. hindawi.com [Internet]. [cited 2023 Jun 2]; Available from: https://www.hindawi.com/journals/bmri/2018/9680514/. 10.1155/2018/9680514 PMC6015709 29984255

[13] Blackstone SR, Nwaozuru U, Iwelunmor J (2017). Factors influencing contraceptive use in sub-saharan Africa: a systematic review. Int Q Community Health Educ.

[14] Okoeguale J, Osagiede EF, Idumwonyi O, Ehigiegba AE. Factors influencing the use of modern contraceptives amongst postpartum women in a rural tertiary hospital in South-South Nigeria. ajol.info [Internet]. 2022 [cited 2023 Jun 2];26(1). Available from: https://www.ajol.info/index.php/ajrh/article/view/224307. 10.29063/ajrh2022/v26i1.2 37585013

[15] Afriyie P, Journal ETTPAM. 2019 undefined. Factors influencing use of modern contraception among married women in ho west district, Ghana: descriptive cross-sectional study. ncbi.nlm.nih.gov [Internet]. [cited 2023 Jun 2]; Available from: https://www.ncbi.nlm.nih.gov/pmc/articles/PMC6620060/. 10.11604/pamj.2019.33.15.17500 PMC6620060 31312331

[16] Kistiana S, Gayatri M, science DSG journal of health., 2020 undefined. Determinants of modern contraceptive use among young married women (age 15–24) in Indonesia. academia.edu [Internet]. [cited 2023 Jun 2]; Available from: https://www.academia.edu/download/71977664/46413.pdf.

[17] Subedi R, Jahan I, Research PBJ. of NH, 2018 undefined. Factors influencing modern contraceptive use among adolescents in Nepal. nepjol.info [Internet]. [cited 2023 Jun 2];16(3). Available from: https://www.nepjol.info/index.php/JNHRC/article/view/21419. 30455481

[18] Asiimwe JB, Ndugga P, Mushomi J, Manyenye Ntozi JP. Factors associated with modern contraceptive use among young and older women in Uganda; a comparative analysis. BMC Public Health. 2014;14(1). 10.1186/1471-2458-14-926 PMC4169837 25195645

[19] Namasivayam A, Lovell S, Namutamba S, Schluter PJ (2020). Predictors of modern contraceptive use among women and men in Uganda: a population-level analysis. BMJ Open.

[20] Wasswa R, Kabagenyi A, Ariho P. Multilevel mixed effects analysis of individual and community level factors associated with modern contraceptive use among married women in Uganda. BMC Public Health. 2021;21(1). 10.1186/s12889-021-11069-0 PMC8314485 34315436

[21] Lete I, Pérez-Campos E (2014). Differences in contraceptive use between Spanish female healthcare providers and Spanish women in the general population aged 23 to 49 years: the HABITS Study. Eur J Contracept Reproductive Health Care.

[22] Solo J, Practice MFGHS. and, 2019 undefined. Provider bias in family planning services: a review of its meaning and manifestations. ghspjournal.org [Internet]. [cited 2023 Jun 5]; Available from: https://www.ghspjournal.org/content/7/3/371?__hstc=175320440.4b44870ec4a577029c49e44b73bd3bee.1615420800131.1615420800132.1615420800133.1&__hssc=175320440.1.1615420800134&__hsfp=1234553885 10.9745/GHSP-D-19-00130 PMC6816811 31515240

[23] Andi J, Wamala R, BOE de la population.…, 2014 undefined. Modern contraceptive use among women in Uganda: An analysis of trend and patterns (1995–2011). ncbi.nlm.nih.gov. [Internet] [cited 2023 Jun 2]; Available from: https://www.ncbi.nlm.nih.gov/pmc/articles/PMC4269974/. 10.11564/28-0-553 PMC4269974 25530666

[24] Hladik W, Stover J, Esiru G, Harper M, Tappero J. The contribution of family planning towards the prevention of vertical HIV transmission in Uganda. PLoS ONE. 2009;4(11). 10.1371/journal.pone.0007691 PMC2766039 19888347

[25] Nsubuga H, Sekandi JN, Sempeera H, Makumbi FE. Contraceptive use, knowledge, attitude, perceptions and sexual behavior among female University students in Uganda: a cross-sectional survey. BMC Womens Health. 2016;16(1). 10.1186/s12905-016-0286-6 PMC4730721 26818946

[26] Abdulahi M, Kakaire O, Namusoke F. Determinants of modern contraceptive use among married Somali women living in Kampala; a cross sectional survey. Reprod Health. 2020;17(1). 10.1186/s12978-020-00922-x PMC7247175 32448285

[27] Rutaremwa G, Kabagenyi A, Wandera SO, Jhamba T, Akiror E, Nviiri HL. Predictors of modern contraceptive use during the postpartum period among women in Uganda: a population-based cross sectional study Health behavior, health promotion and society. BMC Public Health. 2015;15(1). 10.1186/s12889-015-1611-y PMC4372233 25885372

[28] Tetui M, Baroudi M, Ssekamatte T, Birabwa C, Kibira SP, Atuyambe L et al. Total demand, Use and Unmet need for modern contraceptives among women living in Informal settlements in Kira Municipality, Wakiso District, Uganda. Implications for Urban Health. Front Glob Womens Health. 2021;2. 10.3389/fgwh.2021.655413 PMC8593938 34816210

[29] Boadu I. Coverage and determinants of modern contraceptive use in sub-saharan Africa: further analysis of demographic and health surveys. Reprod Health. 2022;19(1). 10.1186/s12978-022-01332-x PMC8781110 35062968

[30] Apanga PA, Kumbeni MT, Ayamga EA, Ulanja MB, Akparibo R. Prevalence and factors associated with modern contraceptive use among women of reproductive age in 20 African countries: a large population-based study. bmjopen.bmj.com [Internet]. 2020 [cited 2023 Jun 7];10:41103. Available from: https://bmjopen.bmj.com/content/10/9/e041103.abstract. 10.1136/bmjopen-2020-041103 PMC7520862 32978208

[31] Ba D, Ssentongo P, Agbese E, Reproductive KKS. 2019 undefined. Prevalence and predictors of contraceptive use among women of reproductive age in 17 sub-Saharan African countries: a large population-based study. Elsevier [Internet]. [cited 2023 Jun 7]; Available from: https://www.sciencedirect.com/science/article/pii/S1877575619300618. 10.1016/j.srhc.2019.06.002 31395230

[32] Sserwanja Q, Musaba MW, Mukunya D. Prevalence and factors associated with modern contraceptives utilization among female adolescents in Uganda. BMC Womens Health. 2021;21(1). 10.1186/s12905-021-01206-7 PMC7877106 33568124

[33] Ahinkorah BO. Predictors of modern contraceptive use among adolescent girls and young women in sub-saharan Africa: a mixed effects multilevel analysis of data from 29 demographic and health surveys. Contracept Reprod Med. 2020;5(1). 10.1186/s40834-020-00138-1 PMC7678092 33292675

[34] Biddlecom A, Awusabo-Asare K, on ABI. perspectives, 2009 undefined. Role of parents in adolescent sexual activity and contraceptive use in four African countries. JSTOR [Internet]. [cited 2023 Jun 4]; Available from: https://www.jstor.org/stable/40233807. 10.1363/ipsrh.35.072.09 19620091

[35] Kabagenyi A, Reid A, JNTPAM. 2016 undefined. Socio-cultural inhibitors to use of modern contraceptive techniques in rural Uganda: a qualitative study. ncbi.nlm.nih.gov [Internet]. [cited 2023 Jun 4]; Available from: https://www.ncbi.nlm.nih.gov/pmc/articles/PMC5324155/. 10.11604/pamj.2016.25.78.6613 PMC5324155 28292041

[36] Cahill N, Sonneveldt E, Stover J, Lancet MWT. 2018 undefined. Modern contraceptive use, unmet need, and demand satisfied among women of reproductive age who are married or in a union in the focus countries of the. Elsevier [Internet]. [cited 2023 Jun 6]; Available from: https://www.sciencedirect.com/science/article/pii/S0140673617331045. 10.1016/S0140-6736(17)33104-5 PMC5854461 29217374

[37] Uganda UNFPA, Uganda MOH. Uganda Family Planning Atlas 2020 [Internet]. X; 2020 [cited 2023 Jun 6]. Available from: https://uganda.unfpa.org/sites/default/files/pub-pdf/uganda_family_planning_atlas.pdf.

[38] Kibira S, Muhumuza C, Bukenya J, one LAP. 2015 undefined. spent a full month bleeding, I thought I was going to die… a qualitative study of experiences of women using modern contraception in Wakiso District, Uganda. journals.plos.org [Internet]. [cited 2023 Jun 6]; Available from: https://journals.plos.org/plosone/article?id=10.1371/journal.pone.0141998. 10.1371/journal.pone.0141998 PMC4629884 26524603

[39] Zimmerman LA, Bell SO, Li Q, Morzenti A, Anglewicz P, Tsui AO. Individual, community and service environment factors associated with modern contraceptive use in five sub-saharan African countries: a multilevel, multinomial analysis using geographically linked data from PMA2020. PLoS ONE. 2019;14(6). 10.1371/journal.pone.0218157 PMC6586288 31220114

[40] Rutaremwa G, AKBpublic. 2015 undefined. Predictors of modern contraceptive use during the postpartum period among women in Uganda: a population-based cross sectional study. bmcpublichealth.biomedcentral.com [Internet]. [cited 2023 Jun 2]; Available from: https://bmcpublichealth.biomedcentral.com/articles/10.1186/s12889-015-1611-y. 10.1186/s12889-015-1611-y PMC4372233 25885372

[41] Andi J, Wamala R, BOE de la population.…, 2014 undefined. Modern contraceptive use among women in Uganda: An analysis of trend and patterns (1995–2011). ncbi.nlm.nih.gov [Internet]. [cited 2023 Jun 4]; Available from: https://www.ncbi.nlm.nih.gov/pmc/articles/PMC4269974/. 10.11564/28-0-553 PMC4269974 25530666

[42] Lakew Y, Reda AA, Tamene H, Benedict S, Deribe K. Geographical variation and factors influencing modern contraceptive use among married women in Ethiopia: evidence from a national population based survey. Reprod Health. 2013;10(1). 10.1186/1742-4755-10-52 PMC3850415 24067083

[43] Stephenson R, Baschieri A, Clements S, Hennink M, Madise N. Contextual influences on modern contraceptive use in sub-Saharan Africa. ajph.aphapublications.org [Internet]. 2007 Jan 7 [cited 2023 Jun 7];97(7):1233–40. 10.2105/AJPH.2005.071522. 10.2105/AJPH.2005.071522 PMC1913073 17538071

[44] health OJA journal of reproductive. 2017 undefined. Determinants of modern contraceptive uptake among Nigerian women: evidence from the national demographic and health survey. ajol.info [Internet]. 2017 [cited 2023 Jun 7];21(3):89. Available from: https://www.ajol.info/index.php/ajrh/article/view/163688. 10.29063/ajrh2017/v21i3.8 29624932

[45] Journal AAPAM. 2021 undefined. Factors influencing the use of modern contraception among reproductive aged women in bangka belitung province, Indonesia. ajol.info [Internet]. 2021 [cited 2023 Jun 2];(39):39. Available from: https://www.ajol.info/index.php/pamj/article/view/219107. 10.11604/pamj.2021.39.39.28870 PMC8356938 34422162

[46] LeMaire WJ. The roman catholic church and contraception. Int J Reprod Contracept Obstet Gynecol [Internet]. 2016 Jun 1 [cited 2023 Jun 9];5(6):2065–9. Available from: https://go.gale.com/ps/i.do?p=HRCA&sw=w&issn=23201770&v=2.1&it=r&id=GALE%7CA457107672&sid=googleScholar&linkaccess=fulltext

[47] Tentler LW. Catholics and Contraception. Catholics and Contraception. 2019.

[48] Ariho P, Kabagenyi A. Age at first marriage, age at first sex, family size preferences, contraception and change in fertility among women in Uganda: analysis of the 2006–2016 period. BMC Womens Health. 2020;20(1). 10.1186/s12905-020-0881-4 PMC6966849 31948426

[49] Maly C,… KMG qualitative, 2017 undefined. Perceptions of adolescent pregnancy among teenage girls in Rakai, Uganda. journals.sagepub.com [Internet]. 2017 Aug 7 [cited 2023 Jun 7];4. Available from: https://journals.sagepub.com/doi/pdf/10.1177/2333393617720555. 10.1177/2333393617720555 PMC5555492 28835911

